# Screening of Key Proteins Affecting Floral Initiation of Saffron Under Cold Stress Using iTRAQ-Based Proteomics

**DOI:** 10.3389/fpls.2021.644934

**Published:** 2021-05-11

**Authors:** Jing Chen, Guifen Zhou, Yan Dong, Xiaodong Qian, Jing Li, Xuting Xu, Huilian Huang, Limin Xu, Liqin Li

**Affiliations:** ^1^Huzhou Central Hospital, Affiliated Hospital of Huzhou Normal University, Huzhou, China; ^2^Huzhou Hospital, Zhejiang University, Huzhou, China; ^3^Department of Chinese Medicine, Zhejiang University of Traditional Chinese Medicine, Hangzhou, China; ^4^Hospital of Chinese Medicine of Changxing County, Huzhou, China

**Keywords:** saffron, flower, cold, iTRAQ, protein

## Abstract

**Background:**

Saffron crocus (*Crocus sativus*) is an expensive and valuable species that presents preventive and curative effects. This study aimed to screen the key proteins affecting the floral initiation of saffron under cold stress and thus increasing yield by regulating the temperature.

**Results:**

Protein expression profiles in flowering and non-flowering saffron buds were established using isobaric tags for relative or absolute quantitation (iTRAQ). A total of 5,624 proteins were identified, and 201 differentially abundant protein species (DAPs) were further obtained between the flowering and non-flowering groups. The most important functions of the upregulated DAPs were “sucrose metabolic process,” “lipid transport,” “glutathione metabolic process,” and “gene silencing by RNA.” Downregulated DAPs were significantly enriched in “starch biosynthetic process” and several oxidative stress response pathways. Three new flower-related proteins, CsFLK, CseIF4a, and CsHUA1, were identified in this study. The following eight key genes were validated by real-time qPCR in flowering and non-flowering top buds from five different growth phases: floral induction- and floral organ development-related genes *CsFLK*, *CseIF4A*, *CsHUA1*, and *CsGSTU7*; sucrose synthase activity-related genes *CsSUS1* and *CsSUS2*; and starch synthase activity-related genes *CsGBSS1* and *CsPU1*. These findings demonstrate the important roles played by sucrose/starch biosynthesis pathways in floral development at the mRNA level. During normal floral organ development, the sucrose contents in the top buds of saffron increased, and the starch contents decreased. In contrast, non-flowering buds showed significantly decreased sucrose contents under cold stress and no significant changes in starch contents compared with those in the dormancy stage.

**Conclusion:**

In this report, the protein profiles of saffron under cold stress and a normal environment were revealed for the first time by iTRAQ. A possible “reactive oxygen species–antioxidant system–starch/sugar interconversion flowering pathway” was established to explain the phenomenon that saffron does not bloom due to low temperature treatment.

## Introduction

*Crocus sativus* L., commonly known as saffron, is a flowering plant in the Iridaceae family that consists of a bulb, white roots, dark green leaves, and flowers with six petals and a sole stigma of three threads that have an intense and unique red color (Rubert et al., 2016). It is a male-sterile triploid lineage that has been propagated vegetatively since its origin and has a relatively stable genotype (Busconi et al., 2015). Iran is one of the most important countries in the world where saffron is produced, and it is widely cultivated in Europe, Asia, Mediterranean countries (especially in the area of Kozani in Greece), and India (Negbi, 1999; Bathaie and Mousavi, 2010). In the last 30 years, saffron has been successfully introduced and cultivated in some areas in China, such as Shanghai, Zhejiang, and Jiangsu (Liu et al., 2017). Saffron is widely used as a natural dietary spice as well as a popular traditional medicine. Several studies have described the health benefits of this plant, such as anticancer activity, antidepressant activity, and cytotoxic effects (Saeed et al., 2010; Kashani et al., 2018; Moradzadeh et al., 2018). Saffron is cultivated for its red stigmatic lobes and blooms only once a year. It is known as the most expensive spice in the world and called “red gold,” and the maximum estimated world production does not exceed 300–400 tons (Kyriakoudi and Tsimidou, 2018). Therefore, increasing the flower number can represent an effective way of producing more saffron to meet the ever-increasing demand in the market (Agayev et al., 2009; Khilare et al., 2019).

For many kinds of flowering bulbs, such as saffron, temperature is one of the most critical factors affecting the formation and development of floral organs. The best temperature range for flower initiation in saffron is from 23 to 27°C, and during the stage of flower primordium formation, flower bud formation rather than leaf bud formation can be inhibited when the ambient temperature is below 16°C. The molecular mechanisms controlling saffron flower formation and development by temperature can be inferred from model plants such as *Arabidopsis*, but validation and further study still need to be carried out. How saffron integrates environmental temperature signals and endogenous signals to ensure the transition from vegetative growth to flowering is of great significance for controlling the number of flowers by regulating the temperature in production practice.

Floral initiation is regulated by a complex genetic network that monitors several environmental signals, which has driven the exploration of genes and proteins that regulate flowering in plants. Light and temperature are important environmental factors affecting the flowering process. Some famous flower genes have been reported to response to the induction of environmental factors and drive flower development. For example, FLOWERING LOCUS T (FT)-like clade induces floral initial process in numerous species by forming regulatory protein complexes with FD-like bZIP transcription factor under a photoperiod pathway (Beinecke et al., 2018). Similarly, the day length-dependent regulation of CONSTANS (CO) protein stability plays a central role in the precise control of flowering time, and it was regulated by GIGANTEA through altering the interaction between F-BOX 1 and ZEITLUPE (Hwang et al., 2019).

The vernalization process is closely related to temperature, in which the expression of many genes and proteins is regulated by temperature. For example, *the flowering locus C* (*FLC*) family, which acts as the key regulator of the vernalization process of *Arabidopsis*, was proven to cooperate with the *VERNALIZATION INSENSITIVE 3* (*VIN3*) family during the course of evolution to ensure a proper vernalization response through epigenetic changes (Kim and Sung, 2013). The studies carried out so far in model plants, including *Arabidopsis* and rice, have allowed extensive knowledge of the flower-related genes and proteins influenced by temperature, and they can serve as a foundational basis for studying the molecular mechanism of temperature regulating in non-model plant flowering. For saffron, the development of flower buds is influenced greatly by temperature, although the possible signaling pathways remain largely unknown. Recently, Haghighi et al. (2020) isolated and characterized a regulator gene involved in saffron floral induction, the *short vegetative phase* (*SVP*) gene, which may repress floral initiation genes in the temperature response pathway.

In our previous studies, full-length transcriptomes of flowering and non-flowering saffron were obtained using a combined next-generation sequencing short-read and single-molecule real-time (SMRT) long-read sequencing approach. Although the genome of the Iridaceae family has not been revealed, several flower-related genes of saffron were identified by transcriptome analysis (Qian et al., 2019). Later, high-quality SMRT sequencing datasets of the full transcriptome for saffron were also presented by Yue et al. (2020). However, the flower development of saffron and the molecular mechanism underlying the flowering response to low temperatures at the protein level have not been reported. In addition, correlations between transcriptomes and proteomes are only in the range of 0.3–0.5 (Voelckel et al., 2010). Therefore, the dynamics of proteomes need further exploration to understand more bioinformation on temperature-responsive flowering in saffron.

Isobaric tags for relative or absolute quantitation (iTRAQ) is one of the most robust and easy-to-use techniques and can be applied in quantitative proteomics of many species. Mass spectrometry technology allows a quantitative comparison of protein abundance by measuring the peak intensities of reporter ions released from iTRAQ-tagged peptides by fragmentation during tandem mass spectrometry (MS/MS) (Karp et al., 2010). It has become a powerful tool in the field of quantitative proteomics and can help with the study of comprehensive protein expression profiles in specific biological responses. For example, Dong et al. (2018) indicated that 4-coumarate-CoA ligase shows a major effect on repressing flavonoid metabolism in chlorotic “Huangjinya” tea leaves by investigating iTRAQ-based quantitative proteomics with phenotypic, biochemical, and transcriptomic data. Here, to understand the underlying mechanism of flowering modulated by temperature, the differentially abundant proteins between flowering plants under normal temperature treatment and non-flowering plants under cold temperature treatment were investigated by iTRAQ-based proteomics and then validated by real-time qPCR. Due to the absence of genomic resources of saffron, the coding sequences were predicted based on the full-length transcriptome sequencing obtained in our laboratory; hence, the accuracy of alignment matches between peptides and assembled transcripts can be improved.

## Materials and Methods

### Plant Materials

**FIGURE 1:**
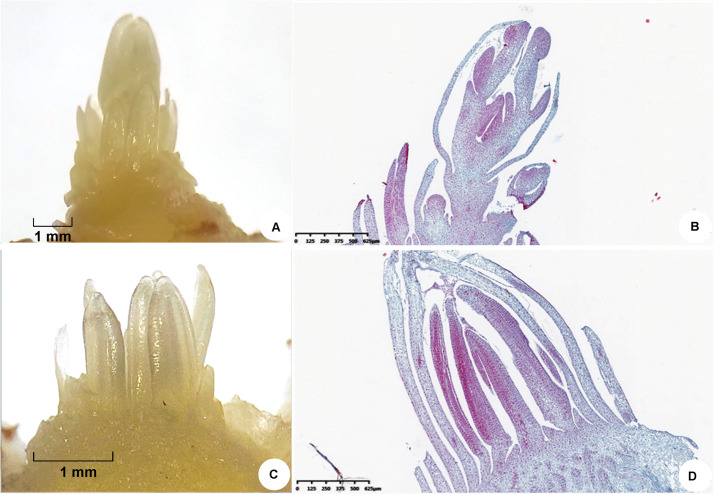
Morphological characteristics of saffron floral organs and leaf organs observed by stereomicroscopy when the top bud length was 7 mm. (**A**,**B**) Floral organs and (**C**,**D**) leaf organs.

Saffron plants which came from the same clone line were cultivated at a research farm at South Tai Lake Agricultural Park, Huzhou (longitude 120.6° E, latitude: 30.52° N, elevation 0 m), using a two-stage cultivation method: corms were planted in soil to allow them to grow outdoors, and they were cultivated indoors without soil (Han et al., 2019). In May 2018, dormant corms (≈25 g) with the same germplasm source and planting method were excavated from the field and divided into two groups, one cultivated at room temperature (20–25°C, flowering phenotype) and the other exposed to low temperature (16°C, non-flowering phenotype) for 30 days. All the samples were exposed to the same humidity and light conditions. When the top bud length was 7 mm and floral organs and leaf organs could be observed clearly by stereomicroscopy, the top buds were collected individually from the two groups (Figure 1). Three replicates were prepared in each group. All samples were frozen in liquid nitrogen and stored at −80°C for iTRAQ and real-time qPCR analysis.

### Protein Extraction

Plant tissues (1 to 2 g) with 10% polyvinylpolypyrrolidone were ground into powder in liquid nitrogen and then sonicated on ice for 5 min in lysis buffer 3 [8 M urea and 40 mM Tris-HCl containing 1 mM phenylmethylsulfonyl fluoride (PMSF), 2 mM ethylenediaminetetraacetic acid (EDTA), and 10 mM dithiothreitol (DTT), pH 8.5] with five volumes of samples. After centrifugation at 25,000 × *g* at 4°C for 20 min, the supernatant was treated by adding five volumes of 10% TCA/acetone with 10 mM DTT to precipitate proteins at −2°C for 2 h overnight. The precipitation step was repeated with acetone alone until there was no color in the supernatant. The proteins were air-dried and resuspended in lysis buffer 3 (8 M urea and 40 mM Tris-HCl containing 10 mM DTT, 1 mM PMSF, and 2 mM EDTA, pH 8.5). Ultrasonication on ice was performed for 5 min (2/3 s) to improve protein dissolution. After centrifugation, the supernatant was incubated at 56°C for 1 h for reduction and alkylated by 55 mM iodoacetamide in the dark at room temperature for 45 min. Five volumes of acetone were added to the samples to precipitate proteins at −20°C for 2 h overnight. Lysis buffer 3 was used to dissolve the proteins with the help of sonication on ice for 5 min.

### Protein Digestion

The protein solution (100 μg) with 8 M urea was diluted four times with 100 mM tetraethylammonium bromide (TEAB). Trypsin Gold (Promega, Madison, WI, United States) was used to digest the proteins at a ratio of protein/trypsin = 40:1 at 37°C overnight. After trypsin digestion, peptides were desalted with a Strata X C18 column (Phenomenex, Torrance, CA, United States) and vacuum-dried according to the manufacturer’s protocol.

### Peptide Labeling

The peptides were dissolved in 30 μl 0.5 M TEAB with vortexing. After the iTRAQ labeling reagents were recovered to ambient temperature, they were transferred and combined with proper samples. Peptide labeling was performed by an iTRAQ Reagent 8-plex Kit according to the manufacturer’s protocol. The labeled peptides with different reagents were combined and desalted with a Strata X C18 column (Phenomenex, Torrance, CA, United States) and vacuum-dried according to the manufacturer’s protocol.

### HPLC Fractionation and LC-MS/MS Analysis

The peptides were separated on a Shimadzu LC-20AB HPLC pump system coupled with a high-pH RP column. The peptides were reconstituted with buffer A (5% ACN, 95% H_2_O, adjusted pH to 9.8 with ammonia) to 2 ml and loaded onto a column containing 5-μm particles (Phenomenex, Torrance, CA, United States). The peptides were separated at a flow rate of 1 ml/min with a gradient of 5% buffer B (5% H_2_O, 95% ACN, adjusted pH to 9.8 with ammonia) for 10 min, 5–35% buffer B for 40 min, and 35–95% buffer B for 1 min. The system was then maintained in 95% buffer B for 3 min and decreased to 5% within 1 min before equilibration with 5% buffer B for 10 min. Elution was monitored by measuring absorbance at 214 nm, and the fractions were collected every 1 min. The eluted peptides were pooled as 20 fractions and vacuum-dried.

Each fraction was resuspended in buffer A (2% ACN, 0.1% FA) and centrifuged at 20,000 × *g* for 10 min. The supernatant was loaded on a Thermo Scientific^TM^ UltiMate^TM^ 3000 UHPLC system equipped with a trap and an analytical column. The samples were loaded on a trap column at 5 μl/min for 8 min and then eluted into a homemade nanocapillary C18 column (ID 75 μm × 25 cm, 3-μm particles) at a flow rate of 300 nl/min. The gradient of buffer B (98% ACN, 0.1% FA) was increased from 5 to 25% in 40 min and then increased to 35% in 5 min, followed by 2-min linear gradient to 80%, maintenance at 80% B for 2 min, then 5% over 1 min, and equilibrated for 6 min.

The peptides separated by nanoHPLC were subjected to tandem mass spectrometry Q EXACTIVE HF X (Thermo Fisher Scientific, San Jose, CA, United States) for data-dependent acquisition detection by nanoelectrospray ionization. The parameters for MS analysis are listed as follows: electrospray voltage, 2.0 kV; precursor scan range, 350–1,500 m/z at a resolution of 60,000 in Orbitrap; MS/MS fragment scan range, >100 m/z at a resolution of 15,000 in HCD mode; normalized collision energy setting, 30%; dynamic exclusion time, 30 s; automatic gain control for full MS target and MS2 target, 3e^6^ and 1e^5^, respectively; and the number of MS/MS scans following one MS scan, 20 most abundant precursor ions above a threshold ion count of 10,000.

### Bioinformatic Pipeline

The raw MS/MS data are converted into MGF format by the corresponding tool, and the exported MGF files are searched by the local Mascot server against the database described above. In addition, quality control is performed to determine if a reanalysis step is needed. IQuant automated software (Wen et al., 2014) was applied to the quantification of proteins. All proteins with a false discovery rate (FDR) less than 1% will proceed with downstream analysis, including Gene Ontology (GO)^1^, euKaryotic Orthologous Groups (KOG) (the eukaryotic KOG set^2^), and Kyoto Encyclopedia of Genes and Genomes (KEGG)^3^. The differentially abundant protein species (DAPs) mentioned below were identified based on the fold change ratio and *Q*-value. Furthermore, DAPs with a fold change ratio > 1.2 and *Q*-value < 0.05 were defined as up-DAPs, while those with a fold change ratio < 0.83 and *Q*-value < 0.05 were defined as down-DAPs.

We also performed deep analyses based on DAPs, including GO enrichment analysis, KEGG pathway enrichment analysis, KOG functional annotation, cluster analysis (heat map), protein interaction analysis (STRING^4^), and subcellular localization analysis (WoLF PSORT^5^). Supplementary Figure 1 presents a list of the workflow for the whole iTRAQ data processing.

### Phylogenetic Analysis

To further understand the possible function of CsFLK, CseIF4a, and CsHUA1, their peptide sequences were aligned with Web BLAST-blastX in the NCBI database for characterization, and the listing protein sequences of other species were downloaded and screened for phylogenetic analysis. The amino acid sequences of CsFLK, CseIF4a, and CsHUA1 were aligned with the selected listing protein sequences using the multiple sequence alignment program ClustalX, and a phylogenetic tree was constructed with MEGA-X software using the neighbor-joining method, with 1,000 bootstrap replicates.

### Quantitative Real-Time Analysis

To complement the changes in abundance at the transcriptional level and validate the key flower proteins, we selected eight candidates between flowering and non-flowering samples cultivated at 20–25 and 16°C. The top buds at 0.1, 0.2, 0.5, 0.7, and 1.1 cm in length from the flowering and non-flowering groups were used to determine different temporal gene expression patterns of genes by qRT-PCR. To determine the expression levels of genes in different tissues, the leaves, corms, roots, pistils, petals, and stamens from flowering samples (top bud lengths of approximately 0.7 cm) were collected. All the samples were ground in liquid nitrogen, and total RNA was prepared using a RNeasy@Plant Mini Kit. The PrimeScript II 1st Strand cDNA Synthesis Kit (TaKaRa, Japan) and SYBR Premix Ex Taq II (TaKaRa, Japan) were used for reverse transcription and qRT-PCR assays. Specific primers of the chosen genes were designed using Primer Premier 5.0 software (Supplementary Table 1). PCR products were verified by dissociation curves, and data were normalized to three endogenous reference genes to obtain ΔCt values. A larger ΔCt value indicated lower gene expression. Water was used as a negative and quality control, and each sample was measured in triplicate.

### Measurement of ROS, Starch, and Sucrose Content

The reactive oxygen species (ROS), starch, and sucrose contents at two developmental stages were measured by ROS ELISA kit (Meibiao Biological, Jiangsu, China), starch content assay kit (Biolab, Beijing, China), and sucrose content assay kit (Biolab, Beijing, China), respectively. The microtiter plate provided in this kit has been pre-coated with an antibody specific to ROS. Standards and samples were added to the appropriate microtiter plate wells. Next, avidin conjugated to horseradish peroxidase was added to each microplate well and incubated. After the 3,3′,5,5′-tetramethylbenzidine substrate solution was added, the enzyme–substrate reaction was terminated by the addition of sulfuric acid solution, and the color change was measured spectrophotometrically (Bio-Rad, Hercules, CA, United States) at a wavelength of 450 nm. The soluble sugar and starch in the sample were separated by 80% ethanol, and the starch was further decomposed into glucose by acid. Finally, by the anthrone colorimetric method, the starch content was determined. To determine the sucrose content, the reducing sugar in the sample was destroyed by alkali first, and then the sucrose was hydrolyzed to glucose and fructose under acidic conditions. Fructose further reacted with resorcinol to form colored substances with characteristic absorption peaks at 480 nm. The absorption values were assayed by a Bio-Rad SmartSpec Plus spectrophotometer (Bio-Rad, Hercules, CA, United States). Three biological replicates for each stage were obtained.

### Statistics

Student’s *t*-test was used to compare statistical significance using SPSS 22.0 software package (SPSS, Chicago, IL, United States), and *p* values < 0.05 were considered significant.

## Results

### Primary Data Analysis and Protein Identification

Differentially accumulated proteins in flowering and non-flowering saffron crocus were identified using the iTRAQ technique, and a total of 836,841 (41,081 matched) spectra were generated. Of these spectra, 20,130 peptides (13,943 unique peptides) and 5,624 proteins were identified with 1% FDR (Supplementary Table 2). Distributions of protein mass, unique peptide number, and peptide length are shown in Supplementary Figure 2. For each identified protein, a greater number of peptides that mapped to the protein indicated higher credibility. Protein sequences covering the ranges 70–100, 60–70, 50–60, 40–50, 30–40, 20–30, 10–20, and below 10% accounted for 0.52, 0.69, 2.58, 7.01, 12.06, 17.39, 28.24, and 31.53% of the total coverage, respectively.

### GO, KEGG, and KOG Functional Classification of All Identified Proteins

Predicting the functions of identified proteins is an important step to fully evaluate or exploit these data. Here the GO, KEGG, and KOG databases were used to annotate and classify all identified proteins. The results of the GO analysis revealed that only a few proteins were located in the extracellular region. In the biological process category, the identified proteins were enriched in “cellular component organization or biogenesis,” “reproduction,” “reproductive process,” and “growth,” suggesting that, at the stage of bud development, saffron was abundant in proteins involved in growth and development. In addition, the proteins were largely enriched in “response to stimulus,” which demonstrated that proteins could play a vital role in adaptation to harmful environments (Supplementary Figure 3A).

In organisms, biological functions are based on a series of protein interactions. KEGG analysis is an effective method that can help to better understand protein biological functions. In the KEGG pathway analysis, a large number of predicted proteins were involved in primary metabolism, such as “carbohydrate metabolism,” “amino acid metabolism,” and “lipid metabolism,” which provide critical compounds needed for growth. Furthermore, the proteins were enriched in “environmental adaptation,” which suggested that various proteins function together in the process of plant arousal response to external stimuli (Supplementary Figure 3B).

Eukaryotic Orthologous Groups were delineated by comparing protein sequences encoded in complete genomes, thus representing major phylogenetic lineages. Each KOG consists of individual orthologous proteins or orthologous sets of paralogs from at least three lineages. Orthologs typically have the same function, thus allowing for the transfer of functional information from one member to an entire KOG. This relation automatically yields a number of functional predictions for poorly characterized genomes. KOGs consist of a framework for functional and evolutionary genome analysis. The KOG functional classification results demonstrated that proteins were strongly enriched in substance and energy metabolism pathways, such as “energy production and conversion,” “carbohydrate transport and metabolism,” “amino acid transport and metabolism,” and “lipid transport and metabolism”; furthermore, many predicted proteins were associated with substance synthesis pathways, such as “translation, ribosomal structure, and biogenesis,” “RNA processing and modification,” and “posttranslational modification, protein turnover, chaperones” (Supplementary Figure 3C). A total of 130 pathway terms were annotated across all identified proteins. The metabolic pathway term was annotated most frequently, and the second most common term was the secondary metabolite synthesis pathway. More detailed data are available in Supplementary Table 3.

Taken together, the bud during this developmental stage is considered the appropriate experimental material because numerous proteins for regulating growth and development are found in the bud. Additionally, the expression changes of resistance-related proteins in cold-treated non-flowering plants further demonstrated the reliability of the determination method in this study.

### Identification and Hierarchical Cluster Analysis of DAPs

In total, 201 proteins, with 120 upregulated and 81 downregulated, were identified as DAPs in the present study (Supplementary Table 4). The top 20 most upregulated and downregulated DAPs are shown in Supplementary Table 5. Among them, non-specific lipid transfer protein (nsLTP, PB.74530.1| m.15978) and glutathione S-transferase U7 (GSTU7; PB.62685.1| m.5798) were upregulated dramatically during the floral process. nsLTP has been reported to be implicated in pollen tube tip growth and fertilization (Chae et al., 2009), and GST protein was involved in the transport of pigment to vacuoles where it accumulates (Momose et al., 2013). In *Arabidopsis*, GSTU7 has a role in seed germination mediated by GSH-ROS homeostasis and ABA signaling (Wu et al., 2020). Thus, nsLTP and GSTU7 proteins play important roles in the development of saffron floral organs, such as the pistil and stamen, where pollen and most pigments are deposited. Compared with cold-treated non-flowering samples, the two most downregulated proteins in normal saffron samples were heat stress transcription factor B-4b (HSFB4B; PB.52694.2| m.74526) and wall-associated receptor kinase 5 (WAK5; PB.32779.1| m.19653). Although the function of class B is less understood, the expression profiles of the HSF B family indicated that they might be integrated into signaling pathways induced by different abiotic stressors, particularly prominent in osmotic, salt, and cold stress samples (Scharf et al., 2012), which suggested that cold stress had a significant effect on the detected protein profiles of saffron. An initial study of *Arabidopsis thaliana* first identified five *WAK* genes (*WAK1*–*WAK5*) that are thought to physically link the extracellular matrix and the cytoplasm and serve a signaling function between them (He et al., 1999). Li et al. (2020) reported that resistance mechanisms engaged in the response to *WAK* signaling include reconstructed homeostasis of ROS species, such as hydrogen peroxide (H_2_O_2_) and superoxide (O_2_^–^). In contrast to WAK1, the function of WAK5 is less reported; however, based on its special transmembrane structure and the relatively high expression levels in cold-treated samples, it may play a role in cell response to cold stress and the production of reactive oxygen species. The possible cause of the increasing expression of HSFB4 and WAK5 in cold-treated non-flowering saffron remains to be further investigated.

### GO, KEGG, and KOG Analysis of DAPs in Saffron

To further determine the promotion or inhibition effect of DAPs on different physiological processes, GO enrichment analysis of upregulated DAPs (relatively highly expressed during the process of floral and leaf organogenesis under ambient temperature) and downregulated DAPs (relatively highly expressed with cold treatment during the process of leaf organogenesis) was carried out.

**FIGURE 2:**
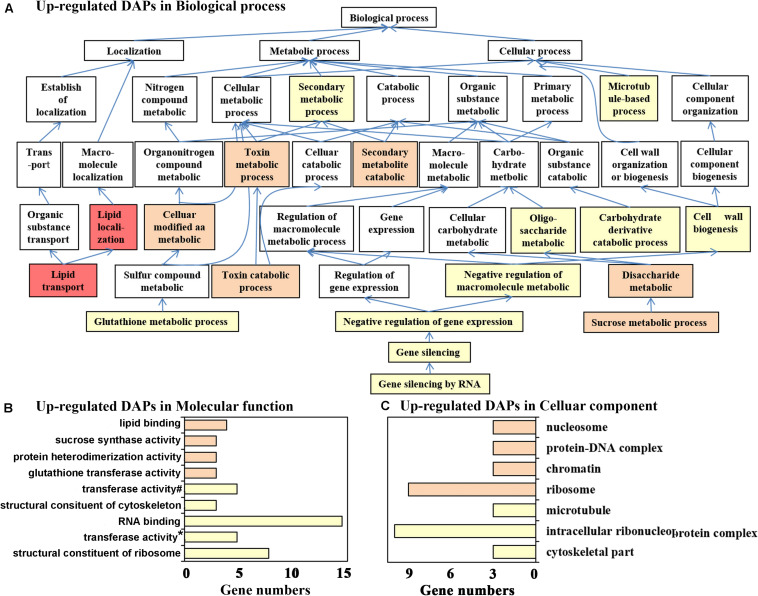
Gene Ontology (GO) annotation for upregulated differentially abundant protein species between flowering and non-flowering saffron crocus. **(A)** Biological process, **(B)** molecular function, and **(C)** cellular component. If multiple GO terms in both molecular function and cellular component are in the same directed acetic graphs, then only the GO term with the lowest level is displayed in the bar chart. Different colors represent different *p*-values: *p* < 1.E-04, red; *p* = 1.E-04–1.E-03, pink; *p* = 0.001–0.01, orange; *p* = 0.01–0.05, yellow; ^#^, transferase activity—transferring glycosyl groups; *, transferase activity—transferring alkyl or aryl (other than methyl) groups.

In total, 120 upregulated DAPs belonged to 142 biological process, 80 molecular function, and 65 cellular component GO terms, while only 18 biological process, 15 molecular function, and 21 cellular component GO terms were significantly enriched, with a *p*-value < 0.05. The lower the level of the GO term, the more specific the functional description, and the most specific and significantly enriched functions are easily detected in the directed acyclic graphs. For biological processes, Figure 2A shows that the most important functions of the upregulated DAPs were “lipid transport,” “sucrose metabolic process,” “glutathione metabolic process,” and “gene silencing by RNA.” Similar molecular functions were also enriched, as shown in Figure 2B, for example, “lipid binding,” “sucrose synthase activity,” “glutathione transferase activity,” and “RNA binding and structural constituent of ribosome.” Correspondingly, upregulated DAPs in flowering samples were significantly located in the cellular components of “nucleosome,” “ribosome,” and “microtubule” (Figure 2C). As previously described, nsLTP (an important lipid transfer protein) and GSTU7 (with glutathione transferase activity) were the top two upregulated proteins, and several lipid transport- and glutamine metabolism-related proteins may be involved in floral organ differentiation and biosynthesis of secondary metabolites of saffron, which are the unique physiological processes only observed in flowering samples. Sucrose not only provides energy for plant growth and development but also plays an important role in signal transduction. Sucrose may function as a key signaling molecule involved in floral transition under normal temperature (Cho et al., 2018). In addition, posttranscriptional gene silencing participates in the regulation of endogenous gene expression in a variety of developmental processes, and increasing attention has been given to RNA silencing during flowering events (Herr et al., 2006; Guo et al., 2016). However, as far as saffron is concerned, related research has not been reported. Inspired by this result, we screened differentially expressed microRNAs between flowering and non-flowering saffron samples to further reveal the flowering mechanism of saffron.

**FIGURE 3:**
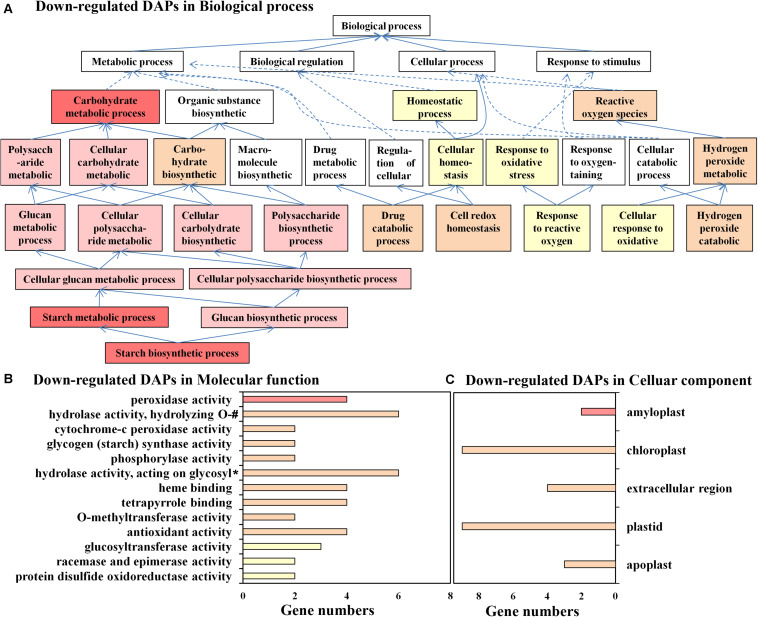
Gene Ontology (GO) annotation for downregulated differentially abundant protein species between flowering and non-flowering saffron crocus. **(A)** Biological process, **(B)** molecular function, and **(C)** cellular component. If multiple GO terms in both molecular function and cellular component are in the same directed acetic graphs, then only the GO term with the lowest level is displayed in the bar chart. Different colors represent different *p*-values: *p* < 1.E-04, red; *p* = 1.E-04–1.E-03, pink; *p* = 0.001–0.01, orange; *p* = 0.01–0.05, yellow; ^#^, hydrolase activity—hydrolyzing *O*-glycosyl compounds; *, hydrolase activity—acting on glycosyl bonds.

A total of 80 downregulated DAPs belonged to 86 biological process, 76 molecular function, and 44 cellular component GO terms. Among them, 18 biological process, 15 molecular function, and 21 cellular component GO terms were significantly enriched with a *p*-value < 0.05. For biological process, “starch biosynthetic process” and several upstream GO terms were significantly enriched (Figure 3A). Interestingly, at least seven downregulated DAPs were annotated as six proteins directly involved in starch biosynthesis: PB.42470.2| m.97279 (glucose-1-phosphate adenylyltransferase large subunit, CsAGPL1), PB.42784.4| m.70714 (ADP-glucose pyrophosphorylase small subunit, CsAGPS1), PB.11585.3| m.159774 (pullulanase 1, CsPULA1), PB.34450.1| m.24905 and PB.33059.2| m.5150 (granule-bound starch synthase 1, CsGBSS1), PB.17182.20| m.162522 (1,4-alpha-glucan-branching enzyme 1, CsSBE1), and PB.1878.6| m.104960 (phosphoglucomutase, CsPGMP), which covered most of the important enzymes in this pathway. A recent study proved that the soluble starch in flower buds of saffron was lower than that in dormancy stages without visible flower organs (Hu et al., 2020). Our results further suggested that the starch content of mixed buds under a normal environment may be lower than that of leaf buds under cold stress and that starch biosynthetic processes may be involved in the response to cold stress and floral induction. The production of ROS and the subsequent oxidative stress response may be the main physiological responses of saffron to cold stress since “cell redox homeostasis,” “response to reactive oxygen,” “cellular response to oxidative,” and “hydrogen peroxide catabolic process” were significantly enriched. This conclusion was also confirmed by ROS measurement results of cold-treated non-flowering top buds and normal flowering top buds collected separately at both the dormant stage (when top bud length < 2 mm) and floral/leaf organogenesis stage (when top length = 6 to 7 mm) (Supplementary Figure 4). PB.58772.3| m.205851 and PB.58772.2| m.185008 were annotated as L-ascorbate peroxidase (CsAPX4), PB.58660.2| m.66725 was annotated as peroxidase 72 (CsPER72), and PB.62862.1| m.30914 was annotated as CsPPX2E. All of them are members of the peroxidase family and can directly catalyze the reduction of hydrogen peroxide to water and alcohol. In addition, several thioredoxin family members (CXXS1, PB.74417.1| m.22999; GRXC2 PB.74995.1| m.182587, and PDIL1 PB.39155.5| m.9557), with the activity of reductase or isomerase to disulfide bond, may also play a role in responding to ROS stress and maintaining cell redox homeostasis.

For molecular function analysis (Figure 3B), the enriched functions, such as “peroxidase activity,” cytochrome-c peroxidase activity,” “antioxidant,” “protein disulfide oxidoreductase activity,” and “glycogen (starch) synthase activity,” further supported the result that the relatively upregulated DAPs under cold stress have significant functions in scavenging oxidants and starch biosynthesis.

In addition, cellular component analysis showed that downregulated DAPs were significantly enriched in amyloplasts and chloroplasts, two important plastids in the top buds of saffron (Figure 3C). The main plastid proteins (CsGBSS1, CsAGPL1, CsAGPS1, and CsPULA1) in DAPs were involved in starch biosynthesis, which occurs in amyloplasts and chloroplasts; the other plastid proteins were involved in carbohydrate metabolic process (PB.23022.16| m.151042, α-glucan phosphorylase) and response to oxidative stress (CsAPX4), which suggested that the normal function of plastids plays an important role in the process of floral initiation.

The KEGG pathway statistics for upregulated and downregulated differentially expressed proteins between the flowering and non-flowering saffron crocus are shown in Supplementary Figure 5A. The results showed that most of the DAPs were involved in biological metabolism and signal transduction-related pathways, thereby demonstrating that they were the main pathways. Specifically, in pathways such as “starch and sucrose metabolism,” “carbon fixation in photosynthetic organisms,” and “pentose phosphate pathway,” more DAPs were upregulated in cold-treated non-flowering saffron. The results suggested that the primary metabolism of the top buds in saffron was significantly changed under cold stress and the distribution of carbon sources in starch and sugar may have changed. In contrast, upregulated DAPs in flowering saffron were enriched in several pathways, such as “nitrogen metabolism,” “isoquinoline alkaloid biosynthesis,” “phenylalanine metabolism,” and “diterpenoid biosynthesis,” suggesting that the biosynthesis and metabolism of nitrogen and secondary metabolites were closely related to flowering. Among all the pathways, “starch and sucrose metabolism,” “stibenoid, diaryheptanoid and gingerol biosynthesis,” “glutathione metabolism,” “galactose metabolism,” and “ribosome biogenesis in eukaryotes” were the most significantly affected (Table 1). More detailed results are available in Supplementary Table 5.

**TABLE 1:** Significantly enriched Kyoto Encyclopedia of Genes and Genomes pathways of differentially expressed proteins.

Pathway	Different proteins with pathway annotation (170)	All proteins with pathway annotation (4,909)	*P*-value	Pathway ID
Starch and sucrose metabolism	17 (10%)	145 (2.95%)	8.245649e-06	ko00500
Stilbenoid, diarylheptanoid, and gingerol biosynthesis	7 (4.12%)	25 (0.51%)	1.497533e-05	ko00945
Glutathione metabolism	8 (4.71%)	86 (1.75%)	0.009336493	ko00480
Galactose metabolism	6 (3.53%)	65 (1.32%)	0.02411393	ko00052
Ribosome biogenesis in eukaryotes	5 (2.94%)	49 (1%)	0.02617775	ko03008

In the functional classification of KOG, DAPs were largely enriched in “carbohydrate transport and metabolism,” “translation, ribosomal structure and biogenesis,” “RNA processing and modification,” and “posttranslational modification, protein turnover, chaperones” (Supplementary Figure 5B). Consistent with the analysis of other bioinformatic analyses, the KOG analysis showed that saffron had vigorous substance metabolism at the bud development stage. More detailed results are provided in Supplementary Table 6.

### Identification of Flower-Related DAPs in Saffron

More than 60 flower-related genes in saffron were reported using PacBio and RNA-sequencing technologies in our previous research. In this study, three new flower-related proteins, CsFLK (flowering locus K homology domain, PB.36509.3| m.13842), CseIF4a (eukaryotic initiation factor 4A-III homolog B, PB.43424.8| m.67369), and CsHUA1 (zinc finger CCCH domain-containing protein 37, PB.42723.2| m.17343), were further identified using iTRAQ technology, and as expected, all of them were significantly upregulated in flowering saffron samples. Lim et al. (2004) demonstrated that *FLK* encodes a putative RNA binding protein with KH motifs that serves as a genetic component of the autonomous flowering pathway and positively regulates flowering by repressing *FLC* expression and posttranscriptional modification. eIF4a is a conserved ATP-dependent RNA helicase and an RNA-dependent ATPase that participates in the initiation of messenger RNA translation. Bush et al. (2016) showed that a pronounced late flowering phenotype appeared in the *eif4a1* mutant because the expression levels of *FLC* and/or other genes in this pathway were regulated, and cell proliferation and growth, which are essential to produce the inflorescence and floral meristems, were retarded. In addition, *eIF4A* has been implicated as a candidate gene for a flowering time quantitative trait locus in maize (Durand et al., 2012). *HUA1* is required for floral determinacy and facilitates pre-mRNA processing of the MADS-box floral homeotic gene *AGAMOUS* in *Arabidopsis* (Irish, 2010). Interestingly, *FLK* was found to be involved in *HUA* activity during C-function maintenance (Rodríguez-Cazorla et al., 2015).

To further understand the possible function of CsFLK, CseIF4a, and CsHUA1, their peptide sequences were aligned with Web BLAST-blastX in the NCBI database to characterize them, and the listing protein sequences were downloaded and screened for phylogenetic analysis. Phylogenetic studies revealed that three proteins of *C. sativus* were intensively clustered with monocotyledon plants, such as *Phoenix dactylifera*, *Elaeis guineensis*, *A. asparagus*, *Oryza sativa*, *Dendrobium catenatum*, and *Phalaenopsis equestris*. Moreover, it also displayed a high bootstrap value with other dicotyledons, and the CsFLK protein showed the highest value, indicating that CsFLK, CseIF4a, and CsHUA1 were relatively conserved proteins. Furthermore, these three proteins were similarly distributed in the branch nearest *to A. asparagus*, which suggested that *C. sativus* was probably closely related to *Asparagus officinalis* in the evolutionary process (Supplementary Figure 6).

### Temporal and Spatial Distribution of Selected DAPs on mRNA Levels

**FIGURE 4:**
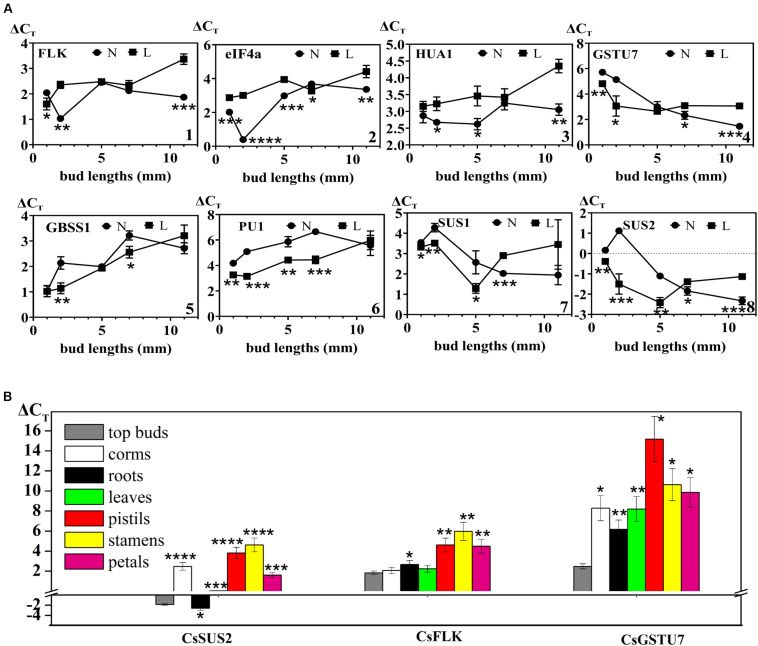
**(A)** Expression levels of genes that might be involved in flowering in different developmental stages between flowering (N) and non-flowering groups (L). (1) *CsFLK* (PB.36509.3| m.13842), (2) *Cs*e*IF4a* (PB.43424.8| m.67369), (3) *CsHUA1* (PB.42723.2| m.17343), (4) *CsGSTU7* (PB.62685.1| m.5798), (5) *CsGBSS1* (PB.34450.1| m.24905), (6) *CsSUS1* (PB.425.38| m.170127), (7) *CsSUS2* (PB.229.41| m.137985), and (8) *CsPU1* (PB.11585.3| m.159774). **(B)** Spatial distribution of three genes (*CsSUS2*, *CsFLK*, and *CsGSTU7*) that might be involved in flowering between flowering and non-flowering groups. Values (mean ± SD) were determined from three independent experiments (*n* = 3). *0.01 ≤ *p* < 0.05; **0.001 ≤ *p* < 0.01; ***0.0001 ≤ *p* < 0.001; ****p < 0.0001. The three genes (*CsSUS2*, *CsFLK*, and *CsGSTU7*) in other tissues were all compared to those in top buds by Student’s *t*-test.

To verify the results of protein quantification at the mRNA expression level and research the expression profiles of 13 key genes selected in this study (including floral induction- and floral organ development-related genes *CsFLK*, *CseIF4A*, *CsHUA1*, and *CsGSTU7*; sucrose synthase activity-related genes *CsSUS1* and *CsSUS2*; and starch synthase activity-related genes *CsGBSS1* and *CsPU1*), another two groups of flowering buds and cold-treated non-flowering buds were collected at five different growth stages: dormancy stage and top bud lengths of 2, 5, 7, and 11 mm. The mRNA expression levels were analyzed by qPCR, and each data point was represented as the mean of three biological replicates. The results showed that the change trends of mRNA and protein levels of all verified genes were consistent (Figure 4A and Supplementary Figure 7). In Figure 4A, three flower-related genes, *CsFLK* (Figure 4A—1), *CseIF4A* (Figure 4A—2), and *CsHUA1* (Figure 4A—3), had higher mRNA levels in flowering buds than in non-flowering buds during the whole developmental process. Interestingly, all of them showed sharply increasing expression levels at the germination stage, when the top buds recovered from dormancy status and growth to 2 mm, while the corresponding non-flowering buds remained unchanged or even declined. The results showed that the *CsFLK, CseIF4A*, and CsHUA1 genes may play important roles in floral induction, and their overexpressed mRNAs may be transcribed into enough flower-related proteins and, subsequently, promote apical meristem differentiation into floral organs. *CsGSTU7* (Figure 4A—4), which was predicted to regulate pigment synthetase activity, showed continuous increases in expression in both flowering and non-flowering buds, which may be due to the need for pigments in both flower and leaf development. However, when the buds grew to 1.1 cm, the development of pistils, stamens, and petals required more pigment synthesis, and *CsGSTU7* showed a significantly higher accumulation in mixed buds of flowers and leaves.

**FIGURE 5:**
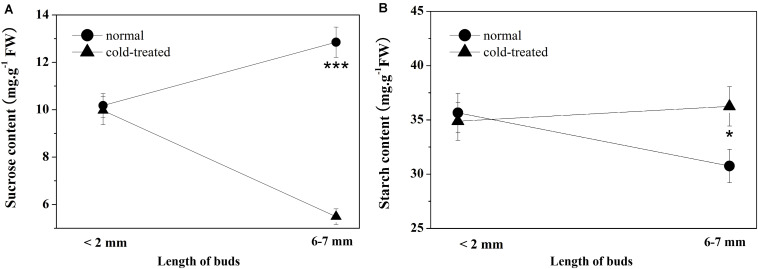
Sucrose and starch contents of apical buds during the flowering transition process between flowering (normal) and non-flowering groups (cold treated). Values (mean ± SD) were determined from three independent experiments (*n* = 3). **(A)** Sucrose content. **(B)** Starch content. *0.01 ≤ *p* < 0.05; **0.001 ≤ *p* < 0.01; ***0.0001 ≤ *p* < 0.001; ****p < 0.0001.

**FIGURE 6:**
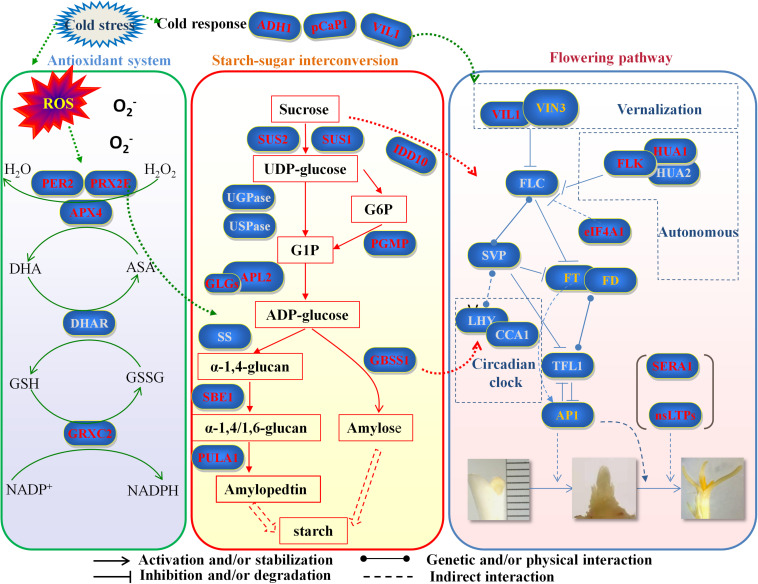
Hypothetical model of “ROS–antioxidant protein–starch biosynthesis–starch/sugar homeostasis–flowering phenotype” to explain the phenomenon that saffron does not bloom due to low temperature treatment. Genes marked in red are identified first in this study. Genes marked in gray are important key genes that have not been identified at present. Genes marked in orange have been reported in other studies in saffron. ROS, reactive oxygen species; PER2, period circadian regulator 2; PRX2F, peroxiredoxin-2F; APX4, L-ascorbate peroxidase; DHAR, dehydroascorbate reductase; GRXC2, glutaredoxin-C2; ADH1, alcohol dehydrogenase 1; pCaP1, plasma membrane-associated cation-binding protein 1; VIL1, VIN3-like protein 1; SUS1, sucrose synthase 1; SUS2, sucrose synthase 1; IDD10, indeterminate domain 10; UGPase, UDP-glucose pyrophosphorylase; USPase, UDP-sugar pyrophosphorylase; PGMP, phosphoglucomutase; APL2, AGPase large subunit; GLGs, glucose-1-phosphate adenylytransferase small subunit; SS, sucrose synthase; GBSS1, granule-bound starch synthase 1; SBE1, 1,4-alpha-glucan branching enzyme; PULA1, pullulanase 1; *VIN3*, *vernalization insensitive 3*; *FLC*, *flowering locus C*; *FLK*, *flowering locus K homology domain*; HUA1, zinc finger CCCH domain-containing protein 37; HUA2, enhancer of AG-4 protein 2; *eIF4A1*, *eukaryotic translation initiation factor 4A1*; *SVP*, *short vegetative phase*; *FT*, *flowering locus T*; *FD*, *flowering locus D*; *LHY*, *late elongated hypocotyl*; *CCA1*, *circadian clock associated 1*; *TFL1*, *terminal flower 1*; *AP1*, *apetala 1*; *SERA1*, *D-3-phosphoglycerate dehydrogenase 1*; nsLTPs, non-specific lipid transfer proteins.

As expected, the expression levels of two key enzyme genes (*CsGBSS1*: Figure 4A—5 and *CsPU1*: Figure 4A—8) in the starch biosynthesis pathway of saffron buds under cold stress were relatively higher than that in normal condition, and both showed continuous declines during the whole flower organ and leaf organ developmental stages. In contrast, the expression levels of *CsSUS1* (Figure 4A—6) and *CsSUS2* (Figure 4A—7) increased continuously from flower bud germination to flower organ formation. Under cold stress, the expression of *CsSUS1* and *CsSUS2* increased in the early developmental stages (bud length = 0.5 cm) but decreased rapidly after the formation of leaf organs. The results suggested that sucrose metabolism-related genes were continuously activated during floral organ development, and compared with normal conditions, cold stress may maintain the activity of starch biosynthesis enzymes and inhibit the activity of sucrose synthase enzymes to some extent at the gene expression levels.

Three genes with gradually accumulated expression during the floral organ formation process (*CsFLK*, *CsSUS2*, and *CsGSTU7*) were further used to detect their distribution profiles at the flowering stage in different tissues, including leaves, corms, roots, pistils, petals, and stamens (Figure 4B). These genes in top buds (length = 7 mm) were detected in the previous section and were also included in this section as a reference. *CsSUS2* showed the highest expression levels in all the tissue organs, followed by *CsFLK* and *CsGSTU7*, which were consistent with the findings for different developmental stages. Except for the expression levels of *CsSUS2* in roots, the expression levels of the three genes in other mature differentiated tissues were lower than those in top buds. In addition, the expression levels of these genes in leaves and roots were significantly higher than those in the pistils, petals, and stamens. How does their significant enrichment in roots and leaves promote the progress of flowering (which is an important physiological process)? Thus, this enrichment needs to be further studied from the tissue distribution of protein levels and the mechanism of protein transport between different tissues.

### Effect of Cold Stress on the Starch and Sucrose Contents in the Top Buds of Saffron

According to the results from the bioinformatic analysis of DAPs and the expression levels of DAPs and their coding genes, cold stress may affect the biosynthesis or metabolism processes of sucrose and starch in the top buds of saffron and then affect the contents of sucrose and starch. Both cold-treated non-flowering top buds and normal flowering top buds were collected separately at both the dormant stage (when top bud length < 2 mm) and floral/leaf organogenesis stage (when top length = 6 to 7 mm). During the normal development of floral organs, the sucrose contents in the top buds of saffron increased from 10.18 ± 0.63 mg g^–1^ (fresh weight) to 12.83 ± 0.69 mg g^–1^, and as expected, the starch contents decreased from 35.66 ± 2.08 mg g^–1^ (fresh weight) to 30.76 ± 1.92 mg g^–1^. In contrast, non-flowering buds showed a significant decrease in sucrose content under cold stress of 5.49 ± 0.33 mg g^–1^, which was almost half of the sucrose content at the dormancy stage. There was no significant change in the starch content of non-flowering buds compared with that in the dormancy stage, while a relatively higher accumulation of starch than the flowering buds was detected, as shown in Figure 5.

## Discussion

### The Model of “ROS–Antioxidant Protein–Starch Biosynthesis–Starch/Sugar Homeostasis–Flowering Phenotype” May Explain the Non-flowering Phenotype of Saffron Top Buds Induced by Cold Stress

Cold stress can have a devastating effect on plant metabolism, disrupt cellular homeostasis, and uncouple major physiological processes, such as alterations in chlorophyll fluorescence, electrolyte leakage, ROS, malondialdehyde, and other metabolites (Liu et al., 2018). Our data suggested that the major result of cold stress-induced cellular changes in the top buds of saffron is the enhanced accumulation of ROS. Basically, upon cold stress, the fluidity of the plasma membrane is altered, and misfolded proteins and excess ROS are produced (Choudhury et al., 2017). If kept unchecked, ROS concentrations will increase in cells and cause oxidative damage to membranes (lipid peroxidation), proteins, and RNA and DNA molecules and can even lead to the oxidative destruction of the cell in a process termed oxidative stress (Mittler, 2002). However, this process is mitigated in cells by a large number of ROS-detoxifying proteins [e.g., superoxide dismutase, ascorbate peroxidase (APX), catalase, glutathione peroxidase, and peroxiredoxin (PRX)]. Indeed a series of peroxidases and thioredoxin (TRX) superfamily members was found to be upregulated under chilling conditions in this study, which can eliminate hydrogen peroxide and maintain redox homeostasis in cells. For example, the members of the peroxidase family, probable L-ascorbate peroxidase 4 (APX4), peroxidase 72 (PER72), and PRX2E, can directly catalyze the reduction of hydrogen peroxide to H_2_O. CXXS1, PDIL, and GRXC2, which are members of the TRX superfamily, have disulfide isomerase and reductase activities. Since both GRXC2 and APX are involved in the glutathione–ascorbate cycle, the results suggested that the glutathione–ascorbate cycle may be the major antioxidant system by which saffron responds to ROS induced by cold stress.

Interestingly, in addition to scavenging ROS, the highly expressed antioxidant proteins induced by cold stress could also influence the expression of enzymes involved in carbohydrate metabolism, which may further affect the normal physiological phenotypes of plants. For example, thioredoxin regulates starch and protein breakdown as a scavenger of H_2_O_2_ in starchy endosperm under oxidative stress (Wong et al., 2003), and sucrose synthase is also regarded as a potential thioredoxin target protein in starchy endosperm of rice. Kim Y. J. et al. (2012) reported that *PDIL1-1* knockout transgenic rice showed an opaque endosperm and a thick aleurone layer and proved that the opaque phenotype of *PDIL1-1* mutant seeds results from the production of irregular starch granules and protein bodies through loss of regulatory activity for various proteins involved in the synthesis of seed components. In our results, the expression of PDIL in top buds under cold stress was 1.8 times higher than that of normal buds, which can potentially affect starch biosynthesis and further starch–sugar homeostasis.

Starch, as a glucose homopolymer, is the major storage carbohydrate deposited in plastids. It can be transported out to cytosol to synthesize sucrose and other disaccharides, which act as a “sugar source” when carbon is needed or as a “sugar sink” in amyloplast when sugars are in excess, which may permit the optimal use of these carbon reserves. Starch–sugar interconversion between plastids and cytosol plays a profound physiological role in all plants (Dong and Beckles, 2019). During the different stages of regular plant growth and development, the ratio of starch–sugar changes dynamically in a reasonable range. More sugar, especially sucrose, may be needed excessively in the flowering transition stage in some plant species (Bernier et al., 1993; Ohto et al., 2001). In this study, compared with cold-treated non-flowering samples, several peptides encoded as SUS1 and SUS2 were significantly upregulated in normal flowering plants, leading to the GO pathway of DAPs enriched in “sucrose metabolic process.” The results were further verified at the mRNA level using more biological replicates, suggesting that the increasing sucrose signal may positively promote the flowering transition of saffron buds. Hu et al. (2020) proved the upregulation of soluble sugar content during saffron floral initiation, and in this study, the sucrose content in the top buds was further detected to increase during the flowering process, which further confirmed this result.

However, when saffron was exposed to cold stress, the balance between starch and sugar was interrupted, as shown by the DAPs enriched in “starch biosynthetic process” in plastids and “sucrose metabolic process” in cytosol. At the metabolic level, the starch contents in flowering buds were significantly decreased, while those in cold-treated non-flowering samples remained almost unchanged, which further confirmed the excessive starch accumulation in the apical buds after cold treatment compared with that under normal conditions. In contrast, the sucrose content sharply decreased compared with normal samples, indicating the positive conversion of sugar to starch. Based on the results in our work, it can be speculated that changes in carbon distribution in the top buds after cold treatment may be responsible for or involved in the loss of the flowering phenotype of saffron. Of course, the changes of carbon distribution in the top buds after cold treatment may be caused by other unknown mechanisms. As previously described, sucrose signaling participates in flower induction. Sugar not only fuels cellular carbon and energy metabolism for the plant but also serves as a signal molecule in coordination with hormone signaling pathways (Rolland et al., 2006) to mediate various plant physiological processes, which likely include flowering and juvenile-to-adult phase transition (Yu et al., 2013). Research has demonstrated that flower induction in plants, which involves sugar levels and responses, may actually be regulated by alterations in sugar flux or sugar signaling (Smeekens et al., 2010). For example, by transporting sugar signaling produced from “source” leaves to the shoot apical meristem, flower development and induction can be determined (Bernier and Périlleux, 2005). Among the various components of sugar, there has been a good amount of evidence suggesting that sucrose promotes flowering in most species that have been examined (Ohto et al., 2001). In our results, proteins were highly enriched in the “starch biosynthetic process,” while the sucrose content significantly decreased in non-flowering saffron under cold treatment. Therefore, the decreased sugar levels induced by excessive starch accumulation may inhibit the initiation signal. On the other hand, the results indicated that some proteins involved in starch synthesis are also regulatory proteins of flowering genes. For example, granule-bound starch synthase (GBSSI), significantly upregulated in non-flowering saffron with cold temperature treatment, is necessary for the synthesis of amylose, and its mRNA expression is controlled by the CCA1 (CIRCADIAN CLOCK ASSOCIATED 1)/LHY (LATE ELONGATED HYPOCOTYL) transcription factors (Tenorio et al., 2003). CCA1/LHY encodes molecular components of the circadian oscillator, which also controls the process of flowering. Coupland’s group found that, under short days, *lhy cca1-1* mutant plants flowered significantly earlier than wild-type plants and further proved that LHY/CCA1 were important transcription regulators of the flowering gene TOC1 (Mizoguchi et al., 2002). Therefore, the overexpression of GBSSI protein in saffron may not only directly cause the accumulation of starch and subsequently starch–sugar redistribution but also indirectly involve the disappearance of the flowering phenotype through the CCA1/LHY-TOC1 axis. Our results provide evidence to show the modulation of starch–sucrose dynamics under cold stress and suggest that this redistribution may cause a change in the flowering phenotype.

In total, saffron under cold stress produced the amount of ROS. In order to eliminate it, the activities of antioxidant proteases were increased significantly and can further influence the expression of enzymes involved in starch biosynthesis. Based on dynamic changes in the starch–sugar interconversion, more starch accumulation in saffron under cold stress may reduce sucrose synthesis. Consequently, low sucrose level and some proteins involved in starch synthesis may inhibit the function of flower-related proteins, which leads to the non-flowering phenotype of saffron. Therefore, we established a hypothetical model—“ROS–antioxidant protein–starch biosynthesis–starch/sugar homeostasis flowering phenotype”—to explain the phenomenon that saffron does not bloom due to low temperature treatment (Figure 6). It will be very challenging and meaningful for us to further explore the detailed molecular mechanism of the pathways.

### Other Proteins That Are Associated With Cold Adaption in Saffron

In addition to antioxidant proteins, a series of other cold-responsive proteins reported in other plants was also highly expressed in the saffron top buds under cold stress, which may facilitate saffron acquisition of tolerance ability. *Arabidopsis* plasma membrane-associated cation-binding protein 2 (PCaP2) is widely expressed in the roots, hypocotyls, cotyledons, root hairs, and pollen tubes (Wang et al., 2007; Kato et al., 2010). Previous studies have demonstrated that PCaP2 is involved in regulating the dynamics of microtubules and F-actin and Ca^2+^ binding ability (Wang et al., 2007; Kato et al., 2010, 2013; Zhu et al., 2013). Interestingly, one work by Kato et al. (2010) found that the mRNA level of PCaP2 increased approximately eightfold by cold treatments. A recent study stated that PCaP2 plays an important and positive function in chilling tolerance and ABA response and can trigger CBF and SnRK2 transcriptional networks (Wang et al., 2018). In this study, PCaP1 (PB.68957.1| m.21748) showed a similar upregulation trend in chilling conditions, which could improve saffron cold tolerance. Classic alcohol dehydrogenase (ADH) is a Zn-binding enzyme that can play important roles in plant biotic and abiotic stress resistance (Komatsu et al., 2011; Pathuri et al., 2011). ADH1 could enhance cold resistance in plants by maintaining the stability of the membrane structure. Additionally, soluble sugars (e.g., sucrose) and amino acids (e.g., asparagine) were found to change accordingly in the adh1 mutant under cold stress (Song et al., 2016). Indeed our data showed that the level of ADH1 (PB.46661.1| m.6484) was significantly upregulated in non-flowering plants under cold temperature treatment, suggesting the important role of ADH1 in maintaining the stability of the saffron cell membrane. More interestingly, VIN3-like protein 1 (VIL1), an important protein involved in the vernalization pathway during the flowering process, was highly expressed in the saffron top buds under cold stress. In most flowering plants experiencing the vernalization pathway, VIL1 mediates the cold response and is involved in modifications to *FLC* and *FLM* chromatin that are associated with an epigenetically silenced state and with acquisition of competence to flower (Sung et al., 2006). Our results showed that the level of VIL1 in saffron was upregulated under cold stress but was unable to promote blooms. This may be related to the fact that saffron blooms in autumn (usually in November) in China and does not share the same vernalization pathway as spring flowering plants.

### Non-specific Lipid Transfer Proteins Are Involved in Flower Induction and Development in Saffron

Plant nsLTPs usually have low molecular mass and are known to play roles in many biological processes, such as cuticular wax synthesis (Kim H. et al., 2012), abiotic stress (Guo et al., 2013), disease resistance (Maldonado et al., 2002; Jung et al., 2009), anther development (Zhang et al., 2010), and pollen tube tip growth (Chae et al., 2009; Chen et al., 2011). Our research showed that at least four peptides (PB.33284.5| m.123278, PB.68442.1| m.210074, PB.8322.7| m.32545, and PB.74530.1| m.15978) that are highly expressed in normal flowering top buds of saffron were annotated as nsLTPs, which suggested that, when flower primordia were formed, the proteins involved in flower organ development began to accumulate, and nsLTPs may play an important role in the differentiation of anthers and pollen tubes of saffron.

In conclusion, we discussed how changes in starch biosynthesis and sucrose metabolism can facilitate adaptive changes in carbon allocation of saffron buds for protection against cold stresses. Stress resistance and flowering are two complex biological processes in plants. The interaction between them is not clear to date, and this study suggested possible interaction pathways between them. In addition, the protein profiles of saffron under cold stress and a normal environment were revealed for the first time. A series of proteins related to flowering and abiotic stress was screened in saffron, and the temporal/spatial distribution of newly identified saffron flower-related genes, including *CsSUS2*, *CsFLK*, and *CsGSTU7*, was explored in this study, which further improved the regulatory network of saffron flowering genes.

## Data Availability Statement

The mass spectrometry proteomics data have been deposited to the ProteomeXchange Consortium *via* the PRIDE (Perez-Riverol et al., 2019) partner repository with the dataset identifier PXD021020.

## Author Contributions

LL designed the experiments. JC and GZ wrote the manuscript. JC, GZ, JL, and XX performed the experiments. HH and LX contributed to the data analysis. YD and XQ contributed to the material planting and sample collection. All the authors read and approved the final manuscript.

## Conflict of Interest

The authors declare that the research was conducted in the absence of any commercial or financial relationships that could be construed as a potential conflict of interest.

## Footnotes

1 http://www.geneontology.org/

2 ftp://ftp.ncbi.nih.gov/pub/COG/KOG

3 http://www.genome.jp/kegg/pathway.html

4 http://string-db.org/

5 https://wolfpsort.hgc.jp/

## Supplementary Material

Supplementary Figure 1 Bioinformatic analysis pipeline.

Supplementary Figure 2 Distributions of protein mass, unique peptide number, and peptide length in this work. Distributions of **(A)** protein mass, **(B)** unique peptide number, and **(C)** peptide length.

Supplementary Figure 3 Gene Ontology (GO), euKaryotic Orthologous Groups (KOG), and Kyoto Encyclopedia of Genes and Genomes (KEGG) annotation for all identified proteins. **(A)** Bar plot of the GO analysis, **(B)** bar plot of the KOG analysis, and **(C)** bar plot of the KEGG analysis.

Supplementary Figure 4 Reactive oxygen species contents of apical buds during the flowering transition process between flowering (normal) and non-flowering groups (cold-treated). Values (mean ± SD) were determined from three independent experiments (*n* = 3). *0.01 ≤ *p* < 0.05; **0.001 ≤ *p* < 0.01; ***0.0001 ≤ *p* < 0.001; *****p* < 0.0001.

Supplementary Figure 5 Kyoto Encyclopedia of Genes and Genomes (KEGG) and euKaryotic Orthologous Groups (KOG) annotation for all the differentially abundant protein species between flowering and non-flowering saffron crocus. **(A)** Bar plot of the KOG analysis and **(B)** bar plot of the KEGG analysis.

Supplementary Figure 6 **(A–C)** Phylogenetic studies of CsFLK, CseIF4a, and CsHUA1. The monocotyledons are marked in red.

Supplementary Figure 7 Expression levels of genes that might be involved in flowering in different developmental stages between flowering (N) and non-flowering groups (L). (1) *CsAGPS1* (PB.42784.4| m.70714), (2) *CsAGPL1* (PB.42470.2| m.97279), (3) *CsPGMP* (PB.1878.6| m.104960), (4) *CsPPX2E* (PB.62862.1| m.30914), and (5) *CsPER72* (PB.58660.2| m.66725).
