# Supplementary figures and images for: Screening of Key Proteins Affecting Floral Initiation of Saffron Under Cold Stress Using iTRAQ-Based Proteomics
Source: Front Plant Sci. 2021 May 11;12:644934. doi: 10.3389/fpls.2021.644934 (PMC8144468; doi:10.3389/fpls.2021.644934)

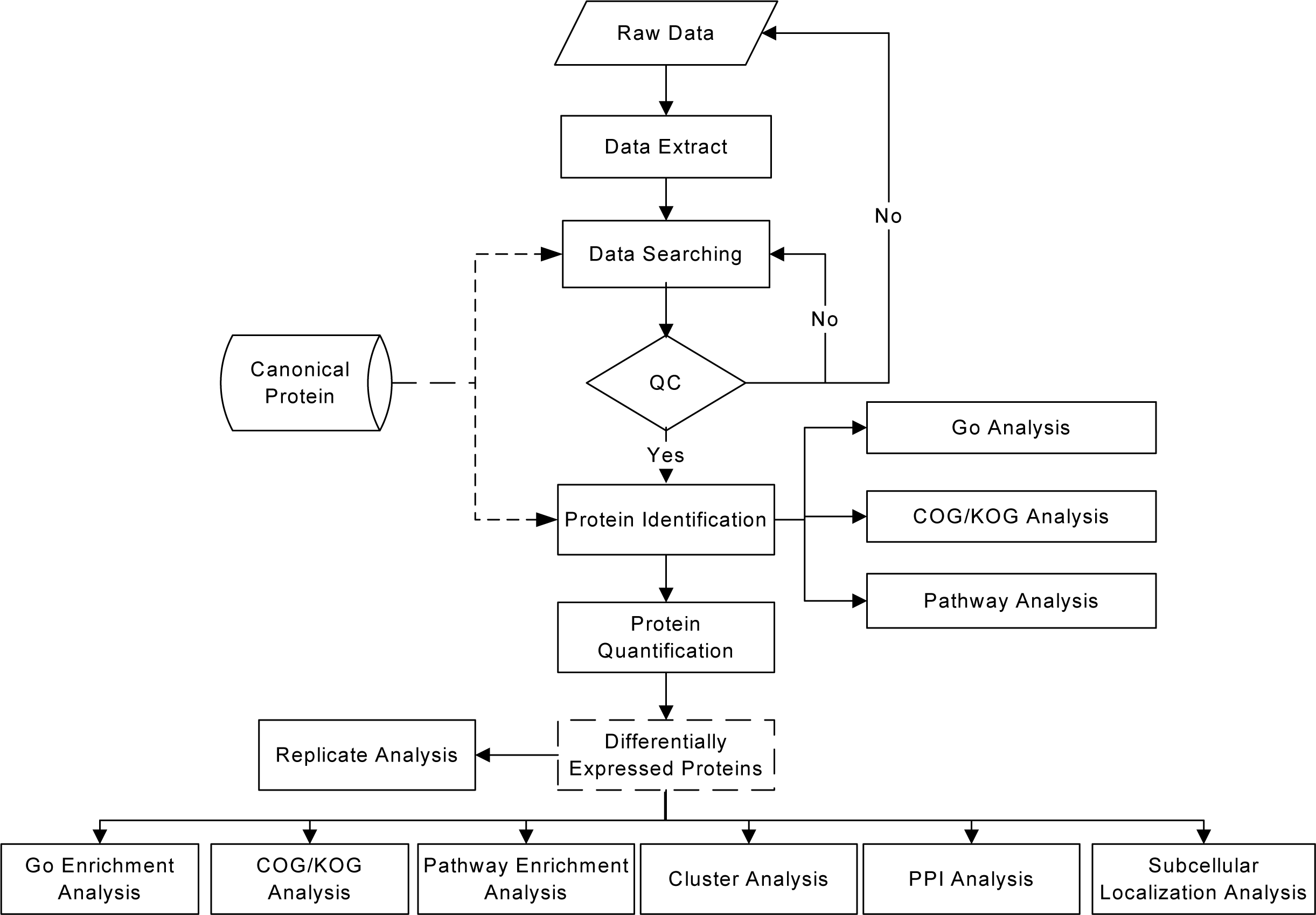

Supplement: Supplementary Figure 1 — Bioinformatic analysis pipeline. [file Image_1.TIF]

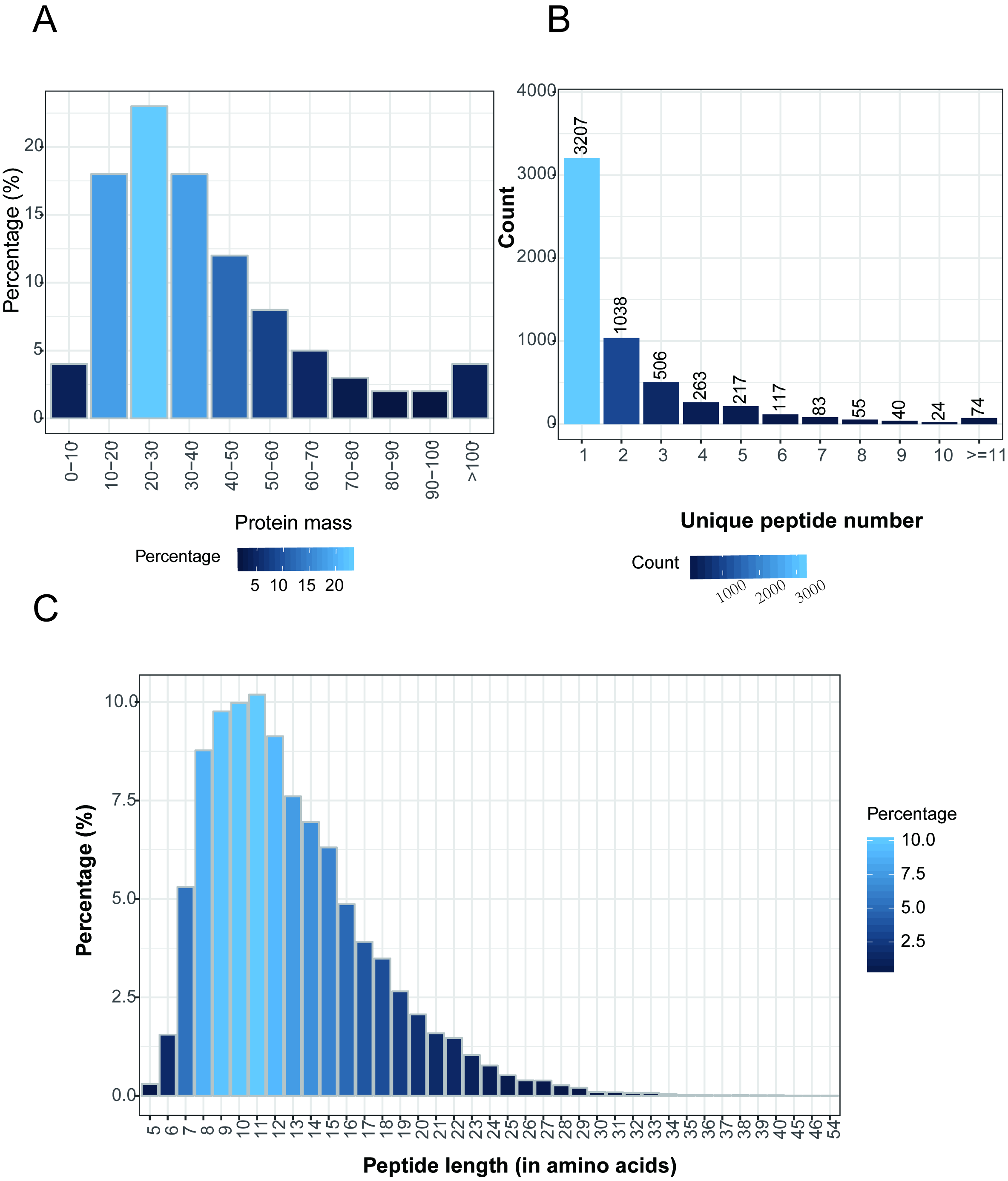

Supplement: Supplementary Figure 2 — Distributions of protein mass, unique peptide number, and peptide length in this work. Distributions of (A) protein mass, (B) unique peptide number, and (C) peptide length. [file Image_2.TIF]

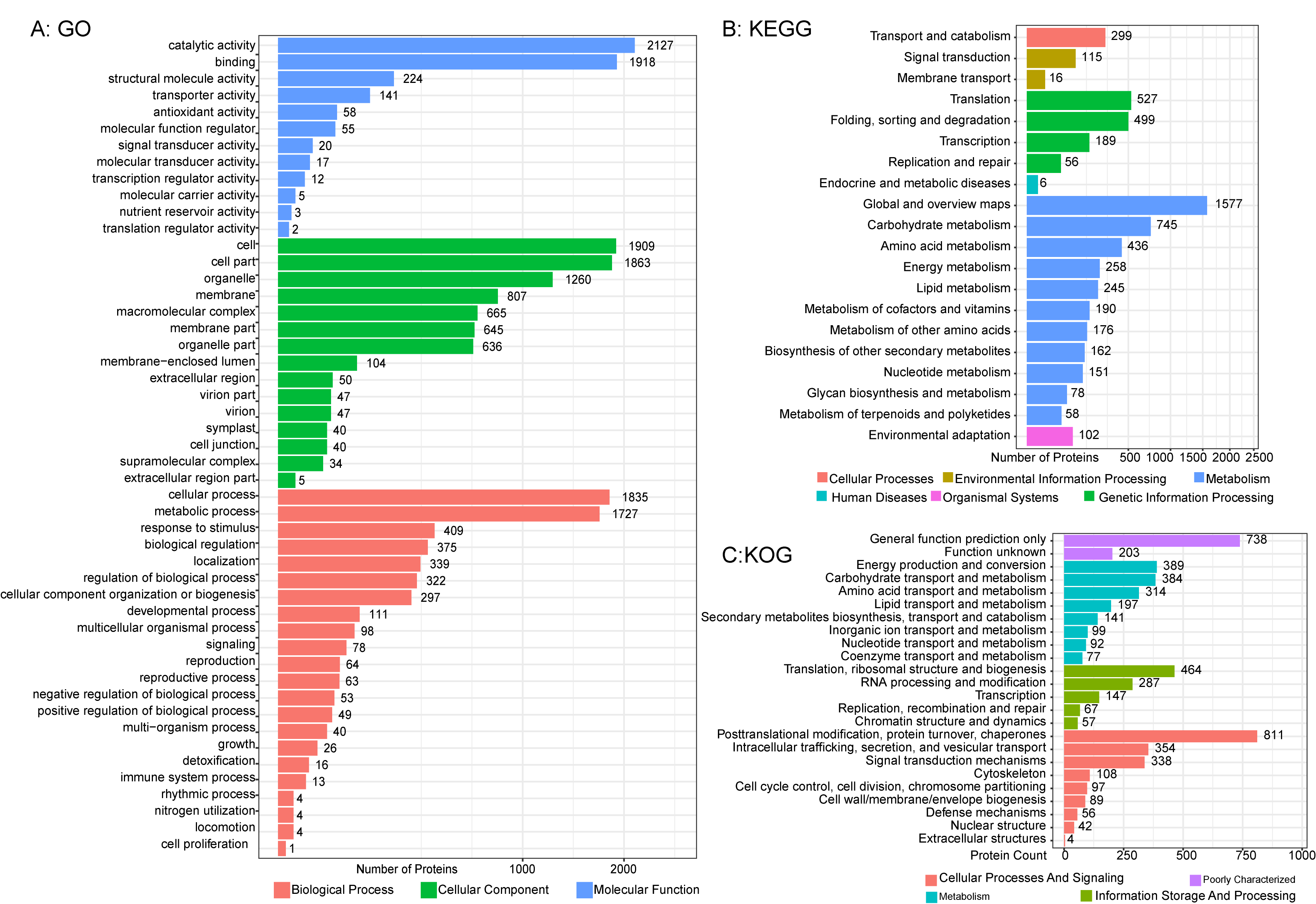

Supplement: Supplementary Figure 3 — Gene Ontology (GO), euKaryotic Orthologous Groups (KOG), and Kyoto Encyclopedia of Genes and Genomes (KEGG) annotation for all identified proteins. (A) Bar plot of the GO analysis, (B) bar plot of the KOG analysis, and (C) bar plot of the KEGG analysis. [file Image_3.TIF]

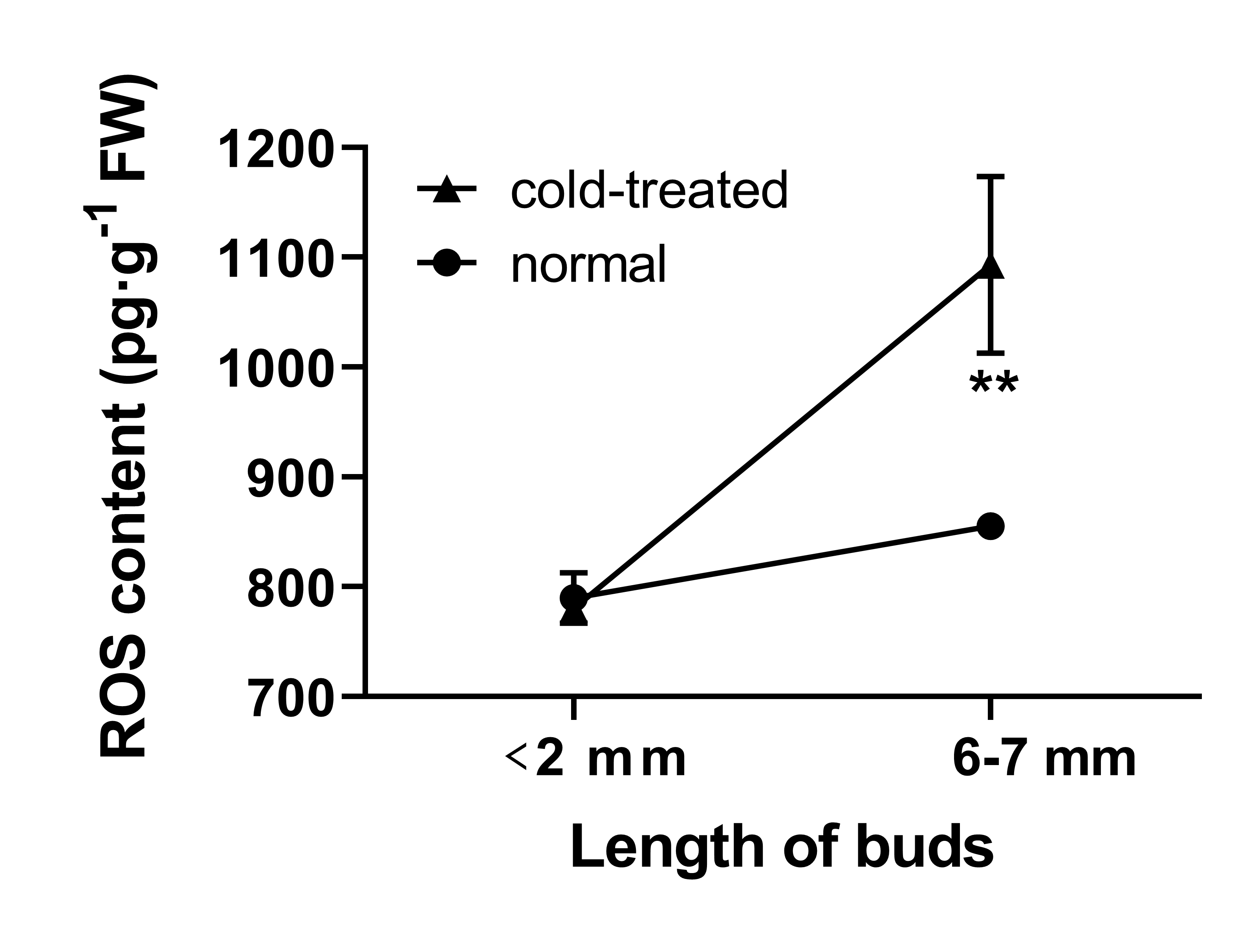

Supplement: Supplementary Figure 4 — Reactive oxygen species contents of apical buds during the flowering transition process between flowering (normal) and non-flowering groups (cold-treated). Values (mean ± SD) were determined from three independent experiments (n = 3). *0.01 ≤ p < 0.05; **0.001 ≤ p < 0.01; ***0.0001 ≤ p < 0.001; ****p < 0.0001. [file Image_4.TIF]

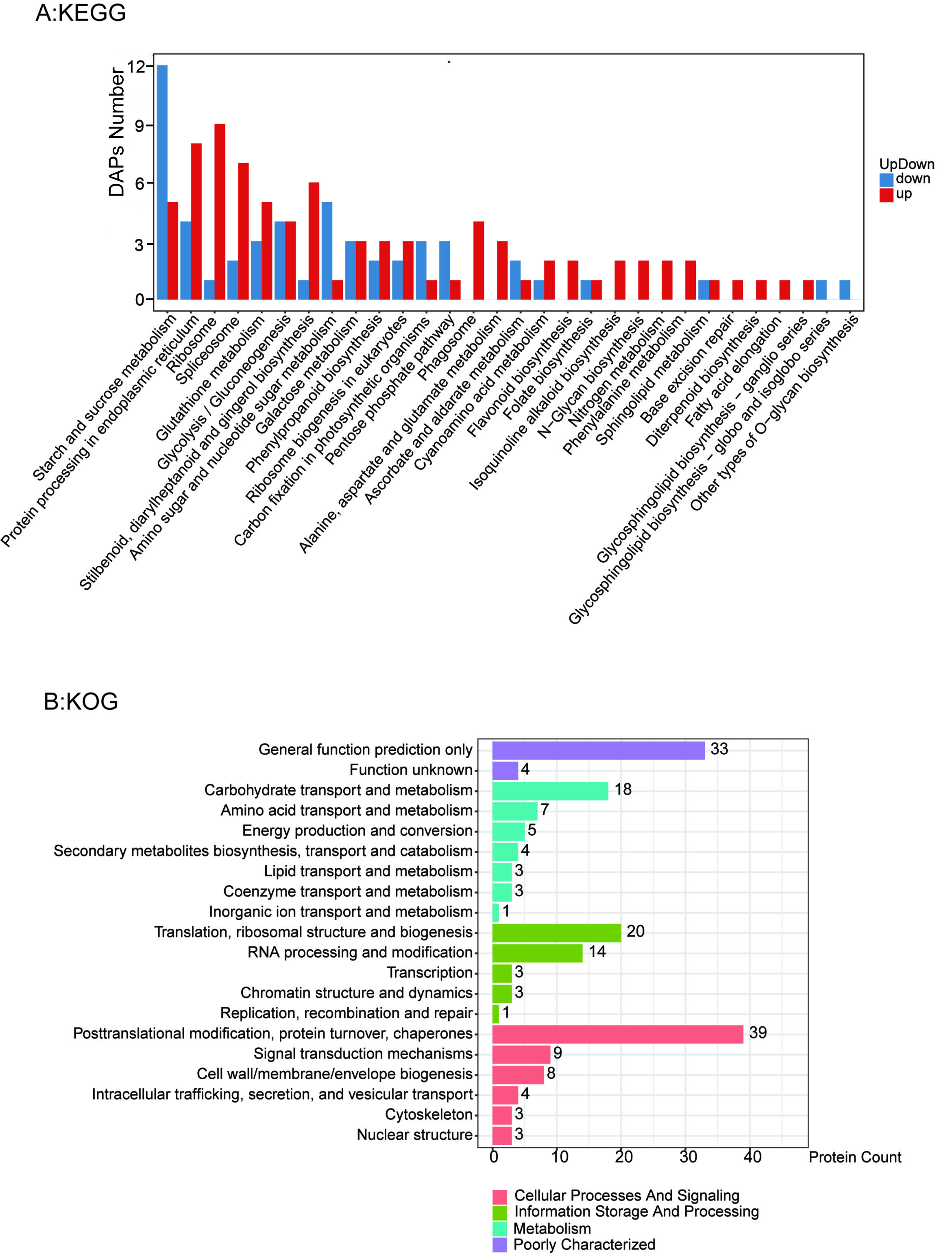

Supplement: Supplementary Figure 5 — Kyoto Encyclopedia of Genes and Genomes (KEGG) and euKaryotic Orthologous Groups (KOG) annotation for all the differentially abundant protein species between flowering and non-flowering saffron crocus. (A) Bar plot of the KOG analysis and (B) bar plot of the KEGG analysis. [file Image_5.JPEG]

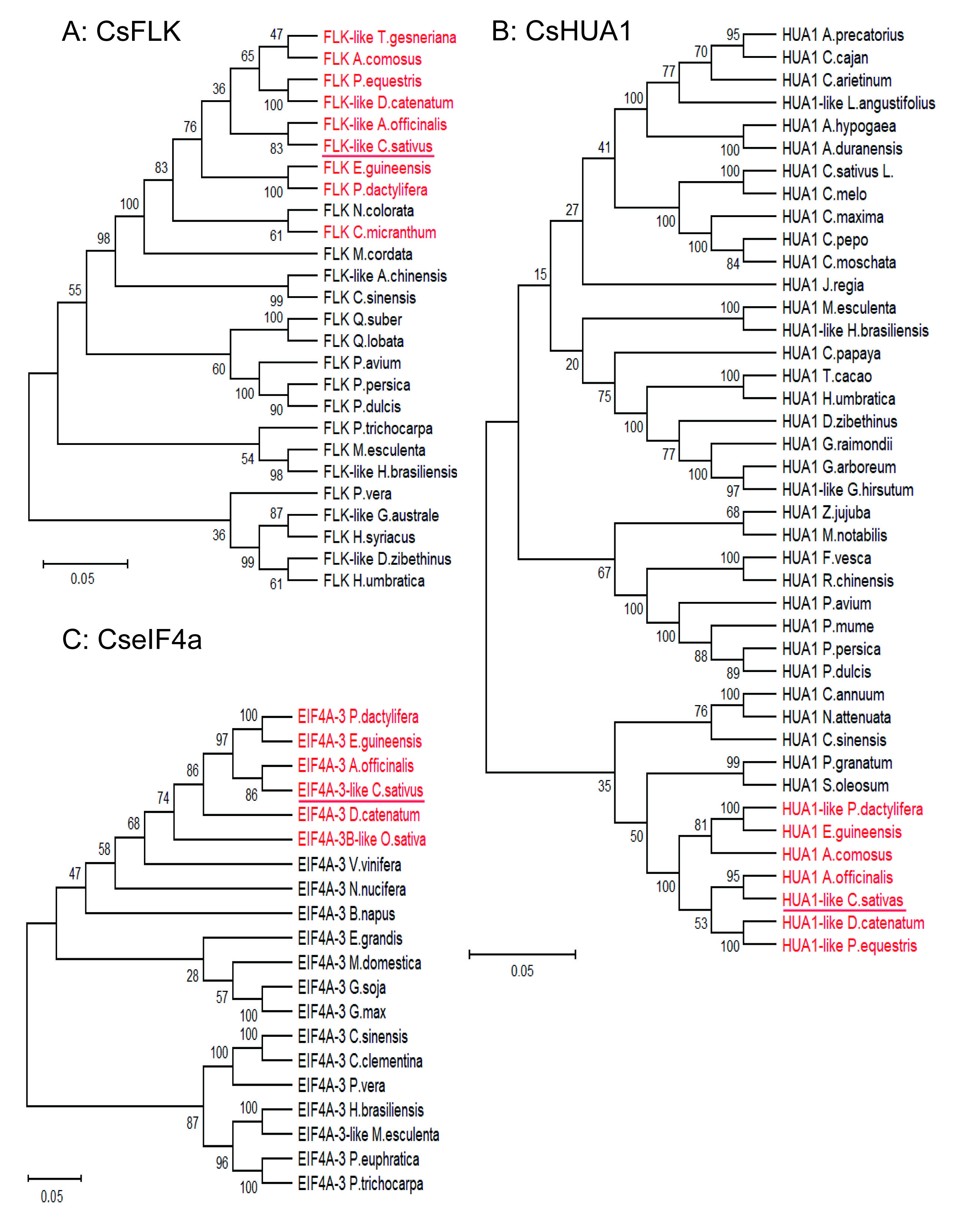

Supplement: Supplementary Figure 6 — (A–C) Phylogenetic studies of CsFLK, CseIF4a, and CsHUA1. The monocotyledons are marked in red. [file Image_6.JPEG]

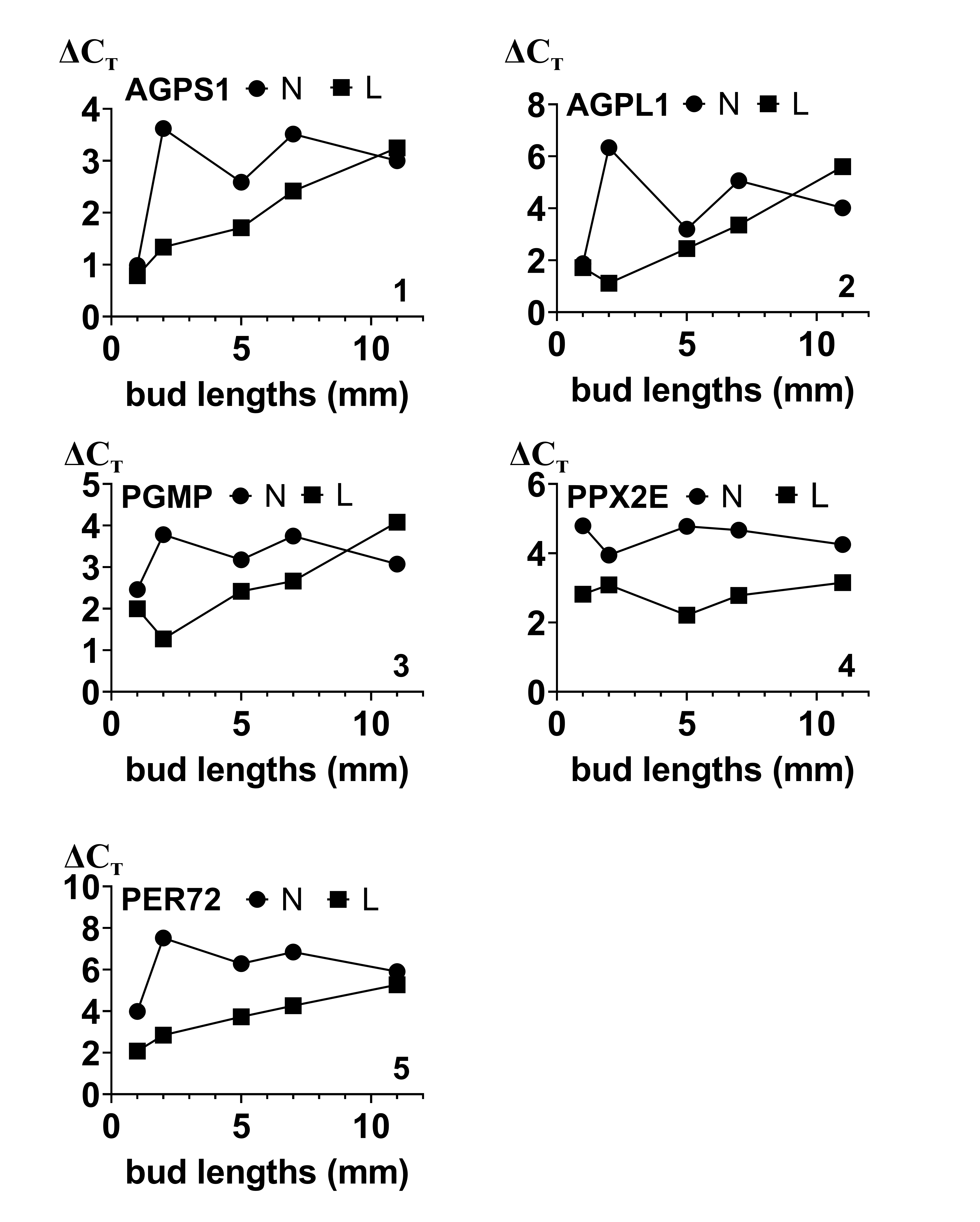

Supplement: Supplementary Figure 7 — Expression levels of genes that might be involved in flowering in different developmental stages between flowering (N) and non-flowering groups (L). (1) CsAGPS1 (PB.42784.4| m.70714), (2) CsAGPL1 (PB.42470.2| m.97279), (3) CsPGMP (PB.1878.6| m.104960), (4) CsPPX2E (PB.62862.1| m.30914), and (5) CsPER72 (PB.58660.2| m.66725). [file Image_7.TIF]
