# Supplementary material for: Screening of Key Proteins Affecting Floral Initiation of Saffron Under Cold Stress Using iTRAQ-Based Proteomics
Source: Front Plant Sci. 2021 May 11;12:644934. doi: 10.3389/fpls.2021.644934 (PMC8144468; doi:10.3389/fpls.2021.644934)
Supplement: Supplementary file 14 [file Table_7.DOCX]

Information regarding statistical data for figures 4A.

| Gene | Bud Lengths(mm) | Group | Mean ΔCт | SD Value | P Value |
| --- | --- | --- | --- | --- | --- |
| FLK | 1 | N | 2.05 | 0.04 | 0.03021 |
|  |  | L | 1.60 | 0.23 |  |
|  | 2 | N | 1.03 | 0.01 | 0.00342 |
|  |  | L | 2.36 | 0.14 |  |
|  | 5 | N | 2.45 | 0.12 | 0.70057 |
|  |  | L | 2.48 | 0.03 |  |
|  | 7 | N | 2.13 | 0.08 | 0.14896 |
|  |  | L | 2.34 | 0.19 |  |
|  | 11 | N | 1.87 | 0.13 | 0.00045 |
|  |  | L | 3.37 | 0.21 |  |
| eIF4a | 1 | N | 2.02 | 0.03 | 0.00034 |
|  |  | L | 2.87 | 0.13 |  |
|  | 2 | N | 0.39 | 0.08 | 0.00001 |
|  |  | L | 3.01 | 0.14 |  |
|  | 5 | N | 2.98 | 0.16 | 0.00078 |
|  |  | L | 3.94 | 0.09 |  |
|  | 7 | N | 3.69 | 0.12 | 0.07446 |
|  |  | L | 3.33 | 0.23 |  |
|  | 11 | N | 3.36 | 0.12 | 0.00874 |
|  |  | L | 4.41 | 0.36 |  |
| HUA1 | 1 | N | 2.87 | 0.21 | 0.13545 |
|  |  | L | 3.15 | 0.15 |  |
|  | 2 | N | 2.67 | 0.04 | 0.01006 |
|  |  | L | 3.23 | 0.20 |  |
|  | 5 | N | 2.62 | 0.17 | 0.01277 |
|  |  | L | 3.46 | 0.30 |  |
|  | 7 | N | 3.25 | 0.20 | 0.41173 |
|  |  | L | 3.42 | 0.26 |  |
|  | 11 | N | 3.05 | 0.17 | 0.00103 |
|  |  | L | 4.35 | 0.20 |  |
| GSTU7 | 1 | N | 5.71 | 0.16 | 0.00286 |
|  |  | L | 4.82 | 0.18 |  |
|  | 2 | N | 5.14 | 0.14 | 0.04182 |
|  |  | L | 3.06 | 0.80 |  |
|  | 5 | N | 3.00 | 0.40 | 0.21402 |
|  |  | L | 2.65 | 0.11 |  |
|  | 7 | N | 2.33 | 0.31 | 0.01748 |
|  |  | L | 3.09 | 0.13 |  |
|  | 11 | N | 1.47 | 0.08 | 0.00015 |
|  |  | L | 3.07 | 0.18 |  |
| GBSS1 | 1 | N | 1.03 | 0.22 | 0.96665 |
|  |  | L | 1.02 | 0.21 |  |
|  | 2 | N | 2.14 | 0.24 | 0.00594 |
|  |  | L | 1.14 | 0.21 |  |
|  | 5 | N | 1.99 | 0.13 | 0.52258 |
|  |  | L | 1.93 | 0.07 |  |
|  | 7 | N | 3.22 | 0.18 | 0.01774 |
|  |  | L | 2.56 | 0.23 |  |
|  | 11 | N | 2.71 | 0.21 | 0.14462 |
|  |  | L | 3.21 | 0.42 |  |
| PU1 | 1 | N | 4.18 | 0.22 | 0.00296 |
|  |  | L | 3.26 | 0.10 |  |
|  | 2 | N | 5.10 | 0.10 | 0.00022 |
|  |  | L | 3.15 | 0.24 |  |
|  | 5 | N | 5.87 | 0.41 | 0.00394 |
|  |  | L | 4.43 | 0.10 |  |
|  | 7 | N | 6.66 | 0.12 | 0.00029 |
|  |  | L | 4.44 | 0.30 |  |
|  | 11 | N | 5.58 | 0.80 | 0.55990 |
|  |  | L | 5.98 | 0.73 |  |
| SUS1 | 1 | N | 3.55 | 0.11 | 0.09536 |
|  |  | L | 3.32 | 0.14 |  |
|  | 2 | N | 4.27 | 0.21 | 0.00459 |
|  |  | L | 3.51 | 0.10 |  |
|  | 5 | N | 2.57 | 0.57 | 0.02259 |
|  |  | L | 1.29 | 0.24 |  |
|  | 7 | N | 2.03 | 0.10 | 0.00088 |
|  |  | L | 2.90 | 0.14 |  |
|  | 11 | N | 1.94 | 0.48 | 0.11666 |
|  |  | L | 3.45 | 1.22 |  |
| SUS2 | 1 | N | 0.16 | 0.05 | 0.00451 |
|  |  | L | -0.39 | 0.16 |  |
|  | 2 | N | 1.12 | 0.08 | 0.00085 |
|  |  | L | -1.50 | 0.50 |  |
|  | 5 | N | -1.11 | 0.12 | 0.00122 |
|  |  | L | -2.42 | 0.25 |  |
|  | 7 | N | -1.85 | 0.20 | 0.03371 |
|  |  | L | -1.39 | 0.15 |  |
|  | 11 | N | -2.33 | 0.19 | 0.00092 |
|  |  | L | -1.14 | 0.14 |  |

Information regarding statistical data for figures 4B.

| Gene | Tssues | Mean ΔCт | SD Value | P Value |
| --- | --- | --- | --- | --- |
| SUS2 | buds | -1.88 | 0.25 | / |
|  | corm | 2.48 | 0.33 | 0.00005 |
|  | roots | -2.61 | 0.45 | 0.06943 |
|  | leaves | 0.04 | 0.02 | 0.00019 |
|  | pistil | 3.81 | 0.47 | 0.00005 |
|  | stamen | 4.63 | 0.67 | 0.00009 |
|  | petal | 1.61 | 0.30 | 0.00010 |
| FLK | buds | 1.84 | 0.20 | / |
|  | corm | 2.07 | 0.25 | 0.27177 |
|  | roots | 2.67 | 0.37 | 0.02746 |
|  | leaves | 2.23 | 0.31 | 0.14073 |
|  | pistil | 4.61 | 0.59 | 0.00155 |
|  | stamen | 5.95 | 0.88 | 0.00137 |
|  | petal | 4.50 | 0.51 | 0.00113 |
| GSTU7 | buds | 2.48 | 0.24 | / |
|  | corm | 8.28 | 1.24 | 0.01241 |
|  | roots | 6.16 | 1.00 | 0.00343 |
|  | leaves | 8.18 | 1.19 | 0.00124 |
|  | pistil | 15.1 | 2.34 | 0.01066 |
|  | stamen | 11.0 | 1.60 | 0.0141 |
|  | petal | 9.88 | 1.43 | 0.01050 |

Information regarding statistical data for figures 5.

| Sbstance | Bud Lengths(mm) | Group | Mean (mg/g FW) | SD Value | P Value |
| --- | --- | --- | --- | --- | --- |
| Sucrose Content | ﹤2 mm | N | 10.2 | 0.44 | 0.84134 |
|  |  | L | 10.1 | 0.60 |  |
|  | 6-7 mm | N | 12.9 | 0.97 | 0.00033 |
|  |  | L | 5.49 | 0.55 |  |
| Starch Content | ﹤2 mm | N | 35.9 | 1.73 | 0.61296 |
|  |  | L | 35.1 | 1.73 |  |
|  | 6-7 mm | N | 31.2 | 1.07 | 0.01356 |
|  |  | L | 35.7 | 1.48 |  |

Information regarding statistical data for supplementary figure 4.

| Bud Lengths(mm) | Group | Mean (pg/g FW) | SD Value | P Value |
| --- | --- | --- | --- | --- |
| ﹤2 mm | N | 789.6 | 23.2 | 0.54581 |
|  | L | 780.4 | 7.3 |  |
| 6-7 mm | N | 855.4 | 3.5 | 0.00694 |
|  | L | 1093.3 | 80.6 |  |
